# Infection of wild-type mice by SARS-CoV-2 B.1.351 variant indicates a possible novel cross-species transmission route

**DOI:** 10.1038/s41392-021-00848-1

**Published:** 2021-12-14

**Authors:** Ting Pan, Ran Chen, Xin He, Yaochang Yuan, Xiaohui Deng, Rong Li, Haiping Yan, Shumei Yan, Jun Liu, Yiwen Zhang, Xiantao Zhang, Fei Yu, Mo Zhou, Changwen Ke, Xiancai Ma, Hui Zhang

**Affiliations:** 1 Institute of Human Virology, Key Laboratory of Tropical Disease Control of Ministry Education, Guangdong Engineering Research Center for Antimicrobial Agent and Immunotechnology, Zhongshan School of Medicine, Sun Yat-sen University, 510080 Guangzhou, Guangdong China; 2 Center for Infection and Immunity Study, School of Medicine, Shenzhen Campus of Sun Yat-sen University, 518107 Shenzhen, Guangdong China; 3 Department of Gastroenterology, The Eighth Affiliated Hospital, Sun Yat-sen University, 518033 Shenzhen, Guangdong China; 4 Sun Yat-sen University Cancer Center, State Key Laboratory of Oncology in South China, Collaborative Innovation Center for Cancer Medicine, 510060 Guangzhou, Guangdong China; 5 Guangdong Provincial People’s Hospital, Guangdong Academy of Medical Sciences, 510080 Guangzhou, Guangdong China; 6 Guangdong Provincial Center for Disease Control and Prevention, 511430 Guangzhou, Guangdong China; 7 National Guangzhou Laboratory, Bio-Island, 510320 Guangzhou, Guangdong China

**Keywords:** Infectious diseases, Infection

## Abstract

COVID-19 is identified as a zoonotic disease caused by SARS-CoV-2, which also can cross-transmit to many animals but not mice. Genetic modifications of SARS-CoV-2 or mice enable the mice susceptible to viral infection. Although neither is the natural situation, they are currently utilized to establish mouse infection models. Here we report a direct contact transmission of SARS-CoV-2 variant B.1.351 in wild-type mice. The SARS-CoV-2 (B.1.351) replicated efficiently and induced significant pathological changes in lungs and tracheas, accompanied by elevated proinflammatory cytokines in the lungs and sera. Mechanistically, the receptor-binding domain (RBD) of SARS-CoV-2 (B.1.351) spike protein turned to a high binding affinity to mouse angiotensin-converting enzyme 2 (mACE2), allowing the mice highly susceptible to SARS-CoV-2 (B.1.351) infection. Our work suggests that SARS-CoV-2 (B.1.351) expands the host range and therefore increases its transmission route without adapted mutation. As the wild house mice live with human populations quite closely, this possible transmission route could be potentially risky. In addition, because SARS-CoV-2 (B.1.351) is one of the major epidemic strains and the mACE2 in laboratory-used mice is naturally expressed and regulated, the SARS-CoV-2 (B.1.351)/mice could be a much convenient animal model system to study COVID-19 pathogenesis and evaluate antiviral inhibitors and vaccines.

## Introduction

Although one year and a half have passed since the initial report of the severe acute respiratory syndrome coronavirus 2 (SARS-CoV-2) infection, the coronavirus disease 2019 (COVID-19) pandemic still persists worldwide.^1^ Many independent dominant SARS-CoV-2 variants, which harbor unique convergent mutations, have emerged locally and eventually circulated worldwide. The lineage B.1.1.7 (also named Alpha) emerged in the UK,^2^ B.1.351 (Beta) emerged in South Africa,^3^ P.1 (Gamma) emerged in Brazil,^4^ B.1.427/B.1.429 (Epsilon) emerged in California of USA, B.1.525 (Eta) emerged in Nigeria,^5^ B.1.526 (Lota) emerged in New York of USA,^6^ B.1.617.1 (Kappa) and B.1.617.2 (Delta) emerged in India,^7^ and C.37 (Lambda) emerged in Peru.^8^ Many mutations lie in the Spike glycoprotein and the corresponding receptor-binding domain (RBD) of these variants, which may compromise Spike- or RBD-based therapeutic antibodies, vaccines, and other countermeasures. Apart from immune evasion, some mutations have been speculated to enable variants to cross-transmit to other animals.

To replicate the symptoms of COVID-19 and evaluate the effectiveness of potential therapeutic interventions, many animal models have been established for preclinical studies.^9^ The original SARS-CoV-2 was able to naturally infect cat, dog, lion, tiger, and mink, as well as experimentally infect monkey, hamster, ferret, and treeshrew, except for house and wild-type mice (*Mus musculus*).^10^ Because the Spike protein of the original SARS-CoV-2 binds tightly to the human angiotensin-converting enzyme 2 (hACE2) but shows no binding affinity to the mouse ACE2 (mACE2), common mice are not susceptible to the original SARS-CoV-2 strain.^11^ Given that the mouse model is a systematic and well-matured platform for in vivo study and quite convenient for the study of viral pathogenesis, two major strategies have been utilized to resolve the above restriction. One is “adapting” virus to mice by serial passaging of virus in mice to enhance the binding affinity of the Spike to mACE2.^12–16^ Another is “adapting” mice to SARS-CoV-2 viruses by introducing hACE2 in mice to facilitate the Spike-binding affinity.^17–23^ However, the mouse-adapted SARS-CoV-2 strains often acquire adaptive mutations, some of which are even within RBD, which may restrict their transmission in humans. Alternatively, the hACE2 protein in hACE2 knock-in mice is ectopically expressed, which may change the tissue tropism of SARS-CoV-2 in vivo. Therefore, both strategies rely on genetic alterations of viral proteins or host receptors, which cannot accurately reflect the symptoms of COVID-19.

A recent report indicated that some mutations within RBD could increase the infectivity of pseudotyped viruses to the cells expressing the ACE2 of a variety of animals including mice.^24^ The pseudotyped viruses with Spike (S) proteins which carried double mutations K417N and N501Y and triple mutations K417N, E484K, and N501Y in RBD exhibited higher infectivity to HEK293T-mACE2 cells in comparison with any single-residue mutants or SARS-CoV-2 (G614). Coincidentally, two recent works showed that mouse-adapted SARS-CoV-2 strains MASCp6 and MASCp36, which harbored single-residue N501Y mutation and triple-residue K417N, Q493H and N501Y mutations, respectively, were capable of infecting wild-type mice.^12,15^ The binding affinities of RBD (N501Y) and RBD (K417N, Q493H, and N501Y) to mACE2 were increased compared with the RBD of SARS-CoV-2 (G614). These works prompted us to investigate whether the natural SARS-CoV-2 variants, especially B.1.351 strain, might acquire the ability to directly infect the wild-type mice. In this report, we demonstrated that the SARS-CoV-2 (B.1.351) is able to replicate with a high titer in lung tissues of young BALB/c and C57BL/6 mice, and induces severe pathological lung lesions and inflammatory responses. B.1.351-infected mice are capable of close-contact transmitting virus to co-housed naive mice. Thus, the infection of SARS-CoV-2 (B.1.351) upon the wild-type mice exhibits a new transmission route. Alternatively, it also provides an animal model for studying SARS-CoV-2 pathogenesis in vivo.

## Results

### Infectivity of pseudotyped SARS-CoV-2 variants to mACE2-expressing cells

**Fig. 1:**
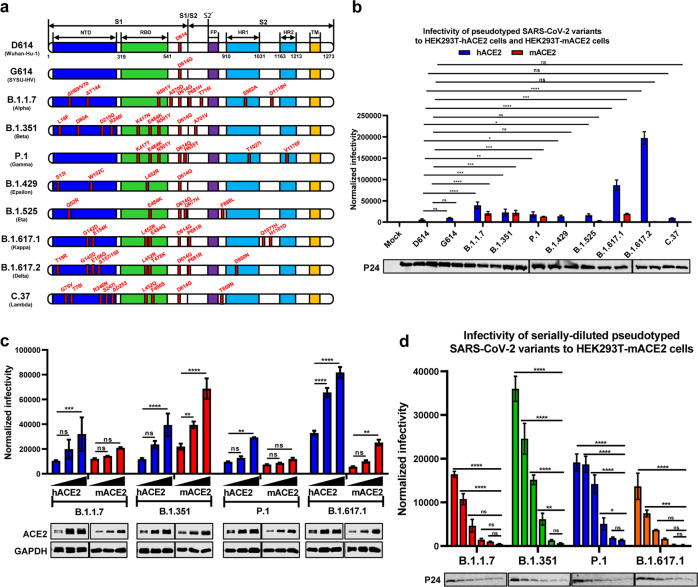
Infectivity of pseudotyped SARS-CoV-2 variants to mACE2-expressing cells. **a** Schematics of the Spike proteins of different SARS-CoV-2 variants which included D614 (Wuhan-Hu-1) virus, G614 (SYSU-IHV), B.1.1.7 (Alpha), B.1.351 (Beta), P.1 (Gamma), B.1.429 (Epsilon), B.1.525 (Eta), B.1.617.1 (Kappa), B.1.617.2 (Delta), and C.37 (Lambda). The mutation sites and types were indicated next to each backbone and written in red. **b** HEK293T-hACE2 and HEK293T-mACE2 cells were incubated with different pseudotyped SARS-CoV-2 viruses, followed by detecting the expression of luciferase at 48 h post infection. The fold changes of luciferase expression were normalized to viral titers followed by normalizing to the mock group and indicated as normalized infectivity (*n* = 3 for each group). The pseudotyped viruses were quantified and normalized with western blot with HIV-1 p24 protein antibody. **c** HEK293T cells were linearly transfected with different amounts (50, 100, and 200 ng in 24-well plates) of hACE2- or mACE2-expressing plasmids, followed by infecting with different pseudotyped SARS-CoV-2 viruses which included B.1.1.7, B.1.351, P.1, and B.1.617.1. The luciferase expression levels were quantified and indicated normalized infectivity. The expression levels of different amounts of hACE2- or mACE2-expressing plasmids transfected HEK293T cells were verified by western blot with antibodies against hACE2 and mACE2. GAPDH proteins were immunoblotted as the internal control (*n* = 3 for each group). **d** The pseudotyped B.1.1.7, B.1.351, P.1, and B.1.617.1 viruses (the initial copies of all the viruses were 2 × 10^5^ copies per μl) were twofold serially diluted and infected HEK293T-mACE2 cells. The normalized infectivity for each variant in each dilution was calculated as in **c** (*n* = 3 for each group). The pseudotyped viruses were quantified with western blot with HIV-1 p24 protein antibody. Data in **b**–**d** represented as mean ± SEM in triplicate. *p* Values were calculated by two-way ANOVA with Dunnett’s multiple comparisons test. ns = *p* ≥ 0.05, *p < 0.05, **p < 0.01, ***p < 0.001, ****p < 0.0001

In order to examine whether some SARS-CoV-2 variants may acquire the ability to infect the mACE2-expressing cells, we constructed two HEK293T cell lines overexpressing hACE2 and mACE2, respectively, after the endogenous hACE2 was knocked out with the CRISPR-CAS9 technique. Meanwhile, we constructed various plasmids expressing the Spike proteins of D614, G614, B.1.1.7, B.1.351, P.1, B.1.429, B.1.525, B.1.617.1, B.1.617.2, and C.37 strains of SARS-CoV-2 (Fig. 1a). These Spike proteins served as envelope proteins to package the pseudotyped SARS-CoV-2 S/IV-1 viruses, which harbor an integrated *luciferase* gene. Upon pseudotyped virus infection, the expression level of luciferase could reflect the viral infectivity.^25^ These different Spike protein-packaged viruses were incubated with both HEK293T-hACE2 and HEK293T-mACE2 cells, which were proceeded to luciferase reporter assays at 48 h post infection. We found that, compared with the D614 variant, a moderate increase of infectivity was observed for all the newly emerged variants in HEK293T-hACE2 cells, except for B.1.617.1 and B.1.617.2 which showed a significant increase in infectivity (*p* < 0.0001) (Fig. 1b). Besides, all the G614-derived variants including the G614 variant itself showed higher infectivity than the D614 virus, which was consistent with the previous finding that D614G-derived variants exhibited higher transmissibility rather than greater pathogenicity in humans and animal models.^26,27^ Surprisingly, we found that, upon infecting HEK293T-mACE2 cells, four variants including B.1.1.7, B.1.351, P.1, and B.1.617.1 showed a significant increase of infectivity compared with D614 variant (*p* < 0.0001 for B.1.1.7, *p* = 0.0006 for B.1.351, *p* = 0.0007 for P.1, *p* < 0.0001 for B.1.617.1) (Fig. 1b). The increased infectivity of B.1.1.7, B.1.351, P.1, and B.1.617.1 to HEK293T-hACE2 and HEK293T-mACE2 cells was linearly correlated with the expression level of hACE2 and mACE2, respectively (Fig. 1c). The positive correlation between viral infectivity and mACE2 expression was more significant for B.1.351 variant (*p* = 0.0066 for medium mACE2 expression group, *p* < 0.0001 for high mACE2 expression group). We also used the serially diluted pseudotyped viruses to infect HEK293T-mACE2 cells. The infectivity of B.1.1.7, B.1.351, P.1, and B.1.617.1 was also positively correlated with the titers of viruses (Fig. 1d). Again, the positive correlation between viral infectivity and viral titers was more significant for B.1.351 variant (*p* < 0.0001, *p* < 0.0001, *p* < 0.0001, and *p* = 0.0065 for the first, second, third, and fourth dilution group, respectively, compared with the highest dilution group). These results suggest that SARS-CoV-2 (B.1.351) could infect mice directly.

### Binding affinities of RBD mutants to hACE2 and mACE2

**Fig. 2:**
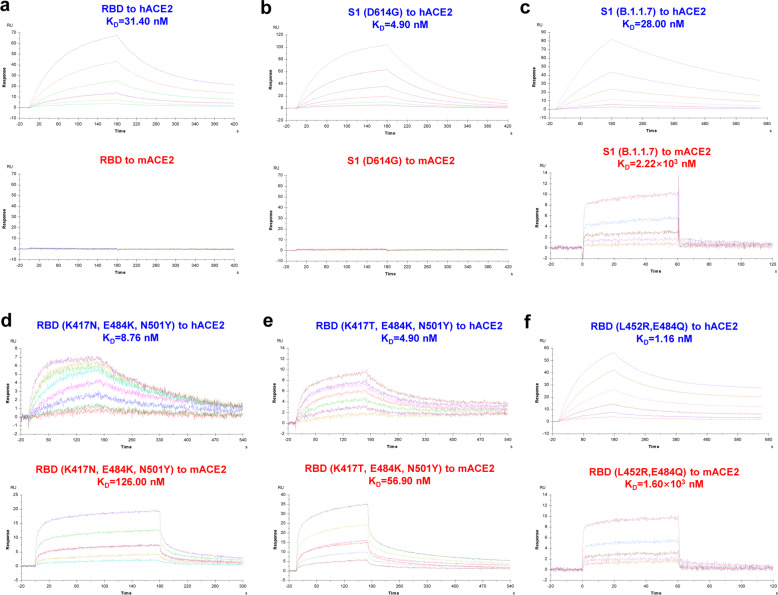
Binding affinities of RBD mutants to hACE2 and mACE2. **a**–**f** The binding affinities of different RBD or S1 mutants to hACE2 and mACE2 were measured by surface plasmon resonance (SPR) assays with a Biacore^TM^ T100 instrument. The *K*_a_, *K*_d_, and *K*_D_ values were measured and calculated by the software BIAevaluation. The *K*_D_ value shown was a mean of three independent experiments. These different RBD or S1 mutants included original RBD (**a**), S1 (D614G) (**b**), S1 (B.1.1.7) (**c**), RBD (K417N, E484K, N501Y) (**d**), RBD (K417T, E484K, N501Y) (**e**), and RBD (L452R, E484Q) (**f**)

To mechanistically search more direct evidence that the Spike proteins of B.1.351, as well as B.1.1.7, P.1, and B.1.617.1 were capable of binding to mACE2, we conducted surface plasmon resonance (SPR) experiments to detect the binding affinities of different RBD or S1 mutants to hACE2 and mACE2. The purified original RBD stood for the RBD of both D614 and G614 variants. We also purified S1 (D614G), S1 (B.1.1.7), S1, and RBD (K417N, E484K, N501Y) of B.1.351, S1, and RBD (K417T, E484K, N501Y) of P.1, S1, and RBD (L452R, E484Q) of B.1.617.1, S1 of B.1.617.2, and S1 of C.37. In addition, we also purified the corresponding receptors hACE2 and mACE2. The purified hACE2 and mACE2 were independently immobilized on sensor chips and incubated with different RBD or S1 mutants. We found that all the RBD mutants showed high binding affinities to hACE2 (binding constant (*K*_D_) ranging from 1.16 to 31.40 nM), whereas the RBD or S1 proteins of D614 and G614 variants showed no binding affinities to mACE2, which was consistent with the previous finding that the original SARS-CoV-2 strains utilized hACE2 as receptor but not mACE2^11^ (Fig. 2 and Supplementary Fig. 1a–c). Interestingly, we found that the S1/RBDs of B.1.1.7, B.1.351, P.1, and B.1.617.1 variants showed high binding affinities to mACE2, the binding constants of which were 2.22 × 10^3^, 126.00, 56.90, and 1.60 × 10^3^ nM, respectively (Fig. 2c–f). The mACE2-binding affinities of RBDs of B.1.351 and P.1 variants were higher than those of B.1.1.7 and B.1.617.1 variants. Meanwhile, we have also found the S1 protein carrying mutations of Delta and Lambda have no binding affinity to mACE2 (Supplementary Fig. 1d, e). Combined with our results from the infectivity assay with pseudotyped viruses and SPR reported here, we believe that the key mutations within various RBD mutants enable Spike proteins to bind with mACE2, which increases the susceptibility of mice to SARS-CoV-2 variants including B.1.1.7, B.1.351, P.1, and B.1.617.1.

### Infectivity and pathogenesis of B.1.351 in wild-type mice

**Fig. 3:**
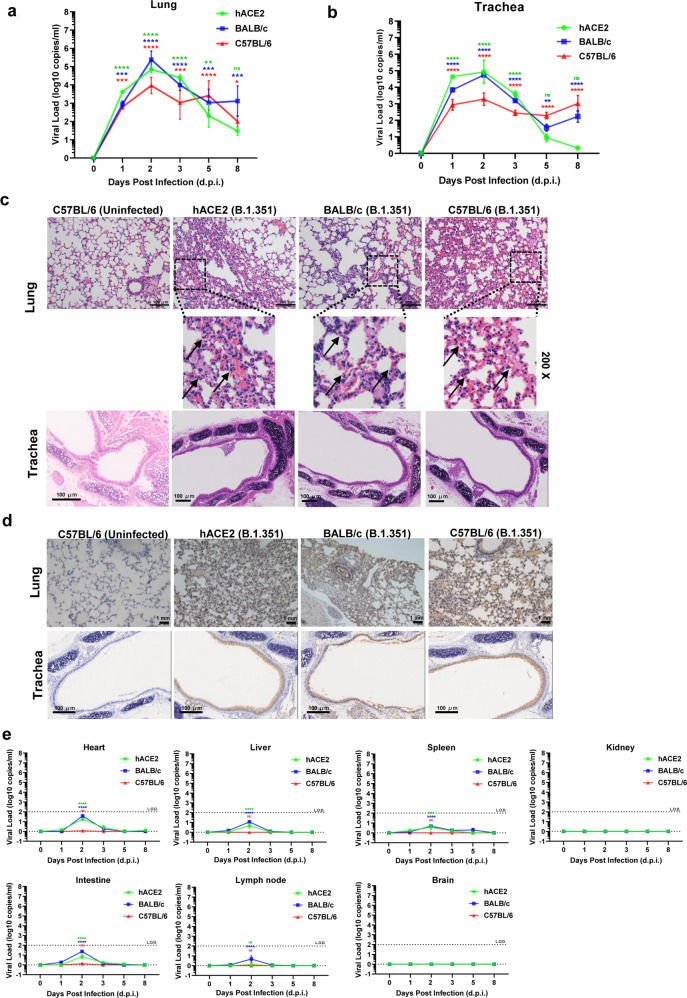
Infectivity and pathogenesis of B.1.351 in wild-type mice. **a** The hACE2 transgenic mice, BALB/c mice, and C57BL/6 mice were intranasally challenged with 5 × 10^5^ FFU of B.1.351 virus. Four C57BL/6 mice were assigned to the uninfected group. Four mice were assigned for each time point in each group. The virus-challenged and uninfected mice were euthanized at 0, 1, 2, 3, 5, and 8 days post infection (d.p.i.). The lungs of each mouse were harvested and homogenized and proceeded to total RNA extraction and RT-qPCR for determining viral RNA copies, which were plotted as log10 copies per ml (*n* = 4). **b** The tracheas of B.1.351-infected hACE2 mice, BALB/c mice, and C57BL/6 mice in each time point were homogenized and proceeded to RNA extraction, followed by RT-qPCR to determine viral copies that were plotted as log10 copies per ml (*n* = 4). **c** The hACE2 transgenic mice, BALB/c mice, and C57BL/6 mice were virus-challenged and euthanized as in **a**. Lungs and tracheas that were harvested on Day 5 were fixed with 4% paraformaldehyde buffer, followed by staining with hematoxylin and eosin (HE). The scale bar in each figure represented 100 μm. Each picture was a representation of four mice. The HE results of lung tissues were 200-fold amplified and shown below the original images. Arrows indicated pathological lung damages. **d** Lungs and tracheas that were harvested on Day 2 were fixed with 4% paraformaldehyde buffer. Then lungs and tracheas of B.1.351-infected and uninfected mice proceeded to immunohistochemical (IHC) assays with antibodies against SARS-CoV-2 Nucleoprotein (N) proteins. The scale bar in each figure represented 1 mm. Each picture was a representation from four mice. **e** The hearts, livers, spleens, kidneys, intestines, lymph nodes, and brains of B.1.351-infected hACE2 mice, BALB/c mice, and C57BL/6 mice in each time point were homogenized and proceeded to RNA extraction, followed by RT-qPCR to determine viral copies that were plotted as log10 copies per ml (*n* = 4). The dotted line indicated the limit of detection (L.O.D.) which was 100 copies per ml. Data in **a**, **b**, **e** are represented as mean ± SEM in quadruplicate. *p* Values were calculated by two-way ANOVA with Dunnett’s multiple comparisons test. Viral load on Days 1, 2, 3, 5, and 8 was compared with viral load on Day 0 for all the mice groups. ns = *p* ≥ 0.05, *p < 0.05, **p < 0.01, ***p < 0.001, ****p < 0.0001

To demonstrate whether B.1.351 could infect wild-type mice, we firstly intranasally challenged C57BL/6 mice with 1 × 10^4^ focus-forming units (FFU), 1 × 10^5^ FFU, and 1 × 10^6^ FFU of B.1.351 virus, respectively. Transgenic hACE2 mice have been widely used to study the pathogenesis of SARS-CoV-2 and immunogenicity of COVID-19 vaccines.^21,28–30^ Thus, B.1.351-infected hACE2 mice (1 × 10^5^ FFU) were also performed as a positive control. Viral copies in the lungs were monitored on day 7 post infection. We found that B.1.351 replicated efficiently and does-dependently in C57BL/6 mice (Supplementary Fig. 2a). However, the body weight loss was not significant and all of the mice were survived on day 7 post infection (*p* > 0.05 for all the body weight changes compared to those on Day 0, except that *p* = 0.0233 for hACE2 mice on Day 2, *p* = 0.0316 for C57BL/6 mice (10^4^ FFU) on Day 7, *p* = 0.0355 for C57BL/6 mice (10^5^ FFU) on Day 7) (Supplementary Fig. 2b, c). Herein, we further intranasally challenged 8-week-old transgenic hACE2 mice, BALB/c mice, and C57BL/6 mice with 5 × 10^5^ FFU of B.1.351 virus. Both BALB/c and C57BL/6 are wild-type mice which are non-genetically modified animals. Viral copies in the lung and other major tissues were monitored on Day 0, 1, 2, 3, 5, and 8 post infection. We found that B.1.351 viruses replicated efficiently in lung and trachea tissues of both BALB/c and C57BL/6 mice besides hACE2 mice (Fig. 3a, b). The viral RNA copies in lungs of each group reached the peak on Day 2 (*p* < 0.0001 for viral load of all the groups compared with those on Day 0), ranging from 1.87 × 10^4^ to 1.24 × 10^5^ copies per ml for hACE2 mice, 9.52 × 10^3^ to 8.77 × 10^5^ copies per ml for BALB/c mice, 7.80 × 10^2^ to 8.00 × 10^4^ copies per ml for C57BL/6 mice (Fig. 3a). Lung tissues of each mouse on Day 5 post infection were proceeded to hematoxylin and eosin (HE) staining. The histopathological analysis showed that the lung tissues of both BALB/c and C57BL/6 as well as hACE2 mice were severely damaged, which were characterized by thickened alveolar septa, collapsed alveoli, interstitial edema, and the infiltration of inflammatory cells (Fig. 3c). Immunohistochemical (IHC) assays with antibodies against SARS-CoV-2 nucleocapsid (N) protein revealed that the lung tissues of virus-challenged mice on Day 2 were diffused with N protein-expressing cells including alveolar epithelial cells and bronchial epithelial cells (Fig. 3d). We also detected high numbers of viral RNA copies and significant pathological changes in the trachea, which was consistent with the previous finding that both human lungs and airways epithelial cells were susceptible to SARS-CoV-2 infection^31^ (Fig. 3b–d). Besides, we detected a slight increase of viral RNA copies in some organs including the heart, liver, and intestines of BALB/_C_ mice, which is consistent with the reports from COVID-19 patients (Fig. 3e).^32–34^ No significant pathological changes were found in all the tissues we examined, although the intestine tissues of B.1.351-infected BALB/c mice were diffused with relatively more N-expressing cells (Supplementary Figs. 3 and 4). The spleen and lymph node exhibited a slight increase of viral RNA copies (<100 copies per ml) (Fig. 3e). However, compared with the lung and trachea, there is no active replication virus in these tissues (Supplementary Fig. 5a, b). These viral RNA copies of these organs only showed on Day 2 and were quickly eliminated on Day 3 or 5.

**Fig. 4:**
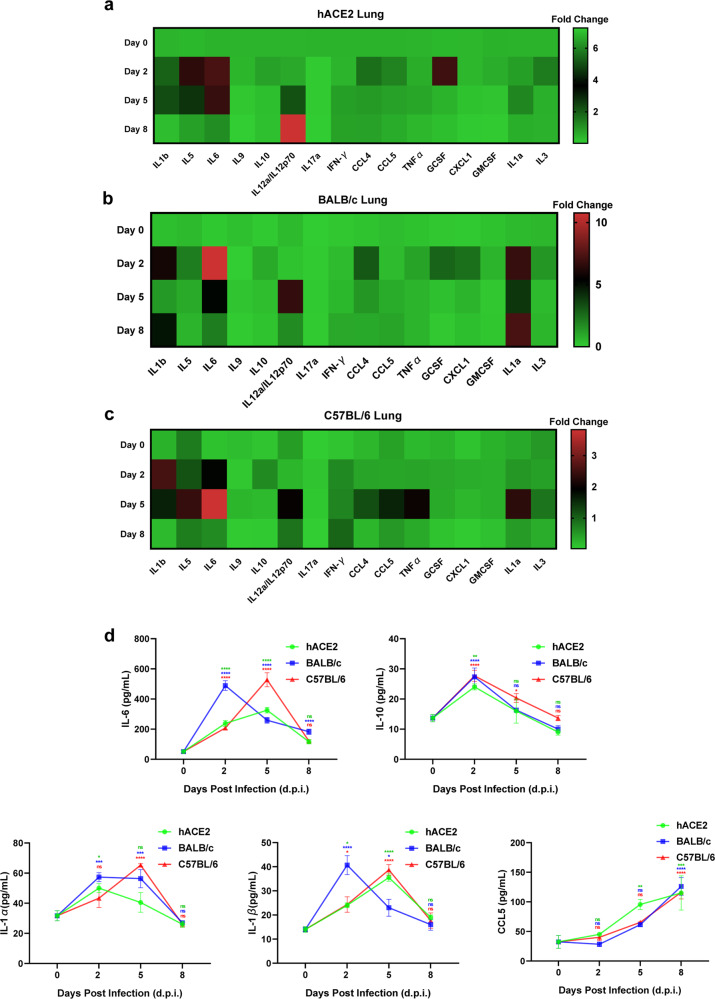
Inflammatory responses in B.1.351-infected mice. **a**–**c** The hACE2 transgenic mice, BALB/c mice, and C57BL/6 mice were challenged with B.1.351 variant as in Fig. 2. The lungs of each mice in each time point were harvested and homogenized. Total RNAs from lungs were extracted and proceeded to RT-qPCR for quantifying the mRNA expression of each cytokine gene, which included *Il1b*, *Il5*, *Il6*, *Il9*, *Il10*, *Il12a/Il12p70*, *Il17a*, *Ifng*, *Ccl4*, *Ccl5*, *Tnfa*, *Gcsf*, *Cxcl1*, *Gmcsf*, *Il1a*, and *Il3*. The RT-qPCR results were normalized to mouse *GAPDH*. The relative expression of each gene was calculated as 2^−ΔΔCt^ method (*n* = 4). **d** The cytokines in sera were also quantified by corresponding ELISA kits, including IL-6, IL-10, IL-1α, IL-1β, and CCL5 (*n* = 4). Data represented as mean ± SEM in quadruplicate. *p* Values were calculated by two-way ANOVA with Dunnett’s multiple comparisons test. ns = *p* ≥ 0.05, *p < 0.05, **p < 0.01, ***p < 0.001, ****p < 0.0001

Previous reports have shown that the cytokines and chemokines were elevated at an early time and are the critical parameters in COVID-19 disease progression, which could provide biomarkers to predict disease progression and targets to develop therapeutic agent.^35^ Thus, we monitored the inflammatory cytokine responses in B.1.351-infected mice. We found that many cytokines messenger RNAs (mRNAs), including *Il1b*, *Il5*, *Il6*, *Il10*, *Il12a/Il12p70*, *Ifng*, *Ccl4*, *Ccl5*, *Tnfa*, *Gcsf*, *Cxcl1*, *Il1a*, and *Il3*, were significantly induced upon B.1.351 infection in the lungs of hACE2 transgenic, BALB/c, and C57BL/6 mice (Fig. 4a–c and Supplementary Table 1). The expression of *Il6* and *Il10* reached the peak on 2 or 5 days post infection (d.p.i.) (*p* < 0.0001 for *Il6* in BALB/c on Days 2 and 5, *p* = 0.0361 for *Il10* in C57BL/6 on Day 2), which was consistent with the previous finding that interleukin (IL)-6 and IL-10 were significantly increased early in severe COVID-19 patients compared with moderate or mild cases.^36–38^ Both *Il1α* and *Il1β* were elevated after 2 d.p.i. (*p* < 0.0001 for *Il1α* in BALB/c on Days 2, 5, and 8, *p* = 0.0002 for *Il1α* in C57BL/6 on Day 5; *p* < 0.0001 for *Il1β* in BALB/c on Days 2 and 8, *p* < 0.0001 and *p* = 0.0138 for *Il1β* in C57BL/6 on Days 2 and 5, respectively), which indicated the infiltration of activated macrophages or neutrophils. The expression of *G-CSF* and *GM-CSF* were also increased upon B.1.351 infection, which promoted the proliferation and maturation of neutrophils, macrophages, and eosinophils (*p* = 0.0087 for *G-CSF* in hACE2 on Day 2, *p* = 0.0004 for *G-CSF* in BALB/c on Day 2; *p* = 0.0223 for *GM-CSF* in BALB/c on Day 2). We also noticed that the expression of *Ccl5* and *Il12a/Il12p70* reached the peak on 5 d.p.i., which could reflect the activation of T and natural killer (NK) cells during viral infection (*p* = 0.0050 for *Ccl5* in C57BL/6, *p* < 0.0001 for *Il12a/Il12p70* in BALB/c and C57BL/6). In addition, we measured some cytokine levels in sera. The cytokine expression patterns of IL-6, IL-10, IL-1α, IL-1β, and C-C chemokine motif ligand 5 (CCL5) in sera were similar to the mRNA expression patterns of these cytokines in lungs, although some cytokines in some mice showed a delayed peak expression (*p* < 0.0001 for IL-6 in hACE2, BALB/c and C57BL/6 on Day 2 and 5; *p* = 0.0015 for IL-10 in hACE2 on Day 2, *p* < 0.0001 for IL-10 in BALB/c and C57BL/6 on Day 2; *p* = 0.0139 for IL-1α in hACE2 on Day 2, *p* = 0.0002 for IL-1α in BALB/c on Day 2, *p* = 0.0004 for IL-1α in BALB/c on Day 5, *p* < 0.0001 for IL-1α in C57BL/6 on Day 5; *p* = 0.0479, *p* < 0.0001 and *p* = 0.0194 for IL-1β in hACE2, BALB/c and C57BL/6 on Day 2, respectively, *p* < 0.0001, *p* = 0.0445 and *p* < 0.0001 for IL-1β in hACE2, BALB/c and C57BL/6 on Day 5, respectively; *p* = 0.0001, *p* < 0.0001 and *p* < 0.0001 for CCL5 in hACE2, BALB/c and C57BL/6 on Day 8, respectively) (Fig. 4d). These results demonstrate that B.1.351 variant is capable of infecting common mice BALB/c and C57BL/6 and inducing significant pathological lung lesions and inflammatory responses, which substantially mimics the infection and pathogenesis of COVID-19 in humans.

### Contact transmission of B.1.351 in wild-type mice

**Fig. 5:**
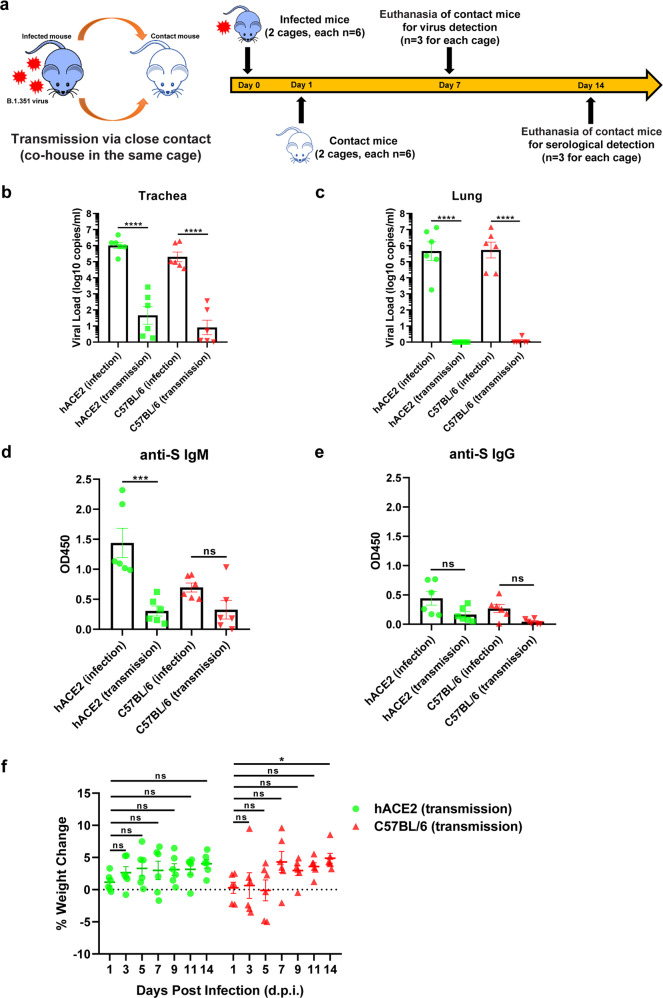
Contact transmission of B.1.351 in wild-type mice. **a** Schematic of experimental design and sample collection of close-contact transmission assay. Twelve C57BL/6 mice were inoculated with 5 × 10^6^ FFU of B.1.351 virus on Day 0 and assigned to two cages (*n* = 6 for each cage). On Day 1 post inoculation, six naive mice were assigned into each cage. On Day 7, three inoculated mice and three contact mice from both cages were euthanized to detect viral RNA copies. On Day 14, three inoculated mice and three contact mice from both cages were euthanized for serological detection. As a control, B.1.351-infected hACE2 mice were co-housed with naive hACE2 mice (6:6 ratio, 2 cages). Viral and serological detections were conducted as above. The body weight changes were monitored every 2 days. **b**, **c** The viral RNA copies of lung and trachea samples from B.1.351-infected and close-contact mice were quantified and represented as log10 copies per ml (*n* = 6). **d**, **e** Reactivity of the serum samples from B.1.351-infected and close-contact mice with SARS-CoV-2 S antigens. Both anti-S IgM and anti-S IgG of these sera were detected. The absorbance at 450 nm represented the relative amount of antibodies (*n* = 6). **f** The body weight changes of both B.1.351-inoculated and close-contact mice. The body weight of each mouse was monitored every 2 days. Data in **b**–**f** are represented as mean ± SEM in sextuplicate. *p* Values in **b**–**e** were calculated by one-way ANOVA with Tukey’s multiple comparison test, which compared the mean of each group with the mean of every other group. *p* Values in **f** were calculated by two-way ANOVA with Dunnett’s multiple comparisons test, which compared the mean of each group with the mean of the first group. ns = *p* ≥ 0.05, *p < 0.05, ***p < 0.001, ****p < 0.0001

To investigate the transmissibility of B.1.351 variant in mice, 12 C57BL/6 mice were placed into 2 cages (*n* = 6 for each cage) and intranasally inoculated with 5 × 10^6^ FFU of B.1.351 virus. On Day 1 post inoculation, 6 naive C57BL/6 mice were co-housed with the inoculated mice (6:6 ratio) to assess the possibility of close-contact transmission (Fig. 5a). As a control, we also co-housed B.1.351-infected hACE2 mice with naive hACE2 mice (6:6 ratio). On Day 7 post inoculation, the trachea and lung samples were collected for virus detection (*n* = 3 for both inoculated and contact mice in each cage). We found that the viral load was detectable in 5 of the 12 trachea samples (10^3.425^, 10^2.286^, and 10^2.786^ copies/ml in hACE2 mice; 10^2^, and 10^2.562^ copies/ml in C57BL/6 mice) and 1 of the 12 lung samples on Day 7 (10^0.4114^ copies/ml in C57BL/6 mouse) (Fig. 5b, c). We also quantified infectious viruses in lung and trachea tissues of these mice by plaque-forming assay. The results showed that the lung and trachea tissues of inoculated mice harbored high titers of infectious viruses (Supplementary Fig. 5c). Some of the lung and trachea tissues of co-housed close-contact transmitted mice also harbored large amounts of infectious viruses (Supplementary Fig. 5c). Although viral RNA copies and infectious viruses of these co-housed contact mice were lower than those of inoculated mice, B.1.351-infected mice were capable of transmitting replication-competent viruses to co-housed naive mice. Serum samples were collected on Day 14 to detect the presence of immunoglobulin M (IgM) and immunoglobulin G (IgG) antibodies which were reactive with SARS-CoV-2 S antigens (*n* = 3 for both inoculated and contact mice in each cage). We found that the IgM value of serum samples was positive while the IgG value was almost negative (Fig. 5d, e). The positive response of anti-S IgM indicated the early immune response to viral infection, which emerged within two weeks. While the anti-S IgG response gradually appeared around 4 weeks post infection, which was later than the specific IgM response. The delayed responses of anti-S IgG in infected mice, especially those in close-contact transmitted mice, also have been reported in another transmissibility study.^39^ Moreover, the body weight loss of inoculated and contact mice has no significant change (Fig. 5f). Based on the RNA copies and the serological analyses of both inoculated and contact mice, we believed that B.1.351-infected mice could transmit the virus to uninfected mice via close contact.

## Discussion

Previous work has indicated that COVID-19 was a zoonotic disease caused by SARS-CoV-2 that jumped from specific animals to humans, although the natural host and intermediate host are still debatable.^10,11^ SARS-CoV-2 seems to cross-transmit within vertebrates such as cat, dog, lion, tiger, mink, monkey, hamster, ferret, treeshrew, and many others.^10,40,41^ Although the original SARS-CoV-2 did not infect mice, we found that SARS-CoV-2 variant B.1.351 acquired the ability to infect mice through enhancing the binding affinity of its RBD to mACE2. Because of the lack of different authentic (live) viruses, we have not tested whether other variants could infect mice. However, we speculate that variants which harbor the same mutations as B.1.351 may also be able to infect mice, such as the P.1 variant which harbors almost the same mutations (501Y, E484K, and K417T) within RBD. We also noticed that both the B.1.1.7 and B.1.617.1 variants were also capable of binding mACE2, albeit the mutations within RBD proteins of both variants were quite different from each other. Specific concerns should be drawn on these mutation hotspots which could enhance the binding affinity of RBD to mACE2.

Our findings provide evidence that the mutation(s) in RBD could become the evolution driving force of zoonotic and anthroponotic transmission of SARS-CoV-2 variants, which could significantly extend the host range among vertebrates. The capability of cross-species transmission of B.1.351 to mice only relied on three mutations within RBD, indicating that RBD mutations among all coronaviruses might lead to the switch or extension of the host range. Interestingly, accumulating evidence has shown that the sera collected from convalescent COVID-19 patients and vaccinated individuals decrease their neutralization capability to B.1.351.^24,42–48^ These shared mutations not only lead to the immune evasion of SARS-CoV-2 variants but also extend the host range to other species, at least the wild-type mice. We have not tested whether B.1.351 could infect other rodents such as wild mice and rats due to the biosafety policy of our BSL-3 facility. While it is very important to further confirm the susceptibility of wild mice and rats to B.1.351 and other SARS-CoV-2 variants. However, the ACE2 receptors of wild mice and rats showed high similarity with wild-type mice.^49,50^ All the inbred mouse strains including C57BL/6, BALB/c, CD-1, and FVB/N, as well as pet or fancy mice, are domesticated from wild house mice (*Mus musculus*). Their mACE2 receptors are almost the same based on homology analysis of ACE2 among selected rodents (99% similarity) (Supplementary Fig. 6). Both mice and rats are members of the *Muridae* family of rodents, the largest family of rodents. Rats and their fleas have been thought to have spread a series of bubonic plague (black death) outbreaks in the fourteenth to the nineteenth century in Europe.^51^ Recently, two reports showed that deer mice, which are members of the *Cricetidae* family of rodents, are also susceptible to SARS-CoV-2 infection and capable of transmitting SARS-CoV-2 to naive deer mice through direct contact.^52,53^ Previous reports also showed that deer mice are natural hosts of both *Borrelia burgdorferi* and Sin Nombre orthohantavirus.^54,55^ Mice including house mice, rats, and deer mice have become the most widely used animals in infection studies. Thus, monitoring coronavirus infection among mice should be brought to the attention to guarantee biosafety. The cross-species transmission of SARS-CoV-2 to rodents such as house mice and deer mice could significantly increase the burden to eliminate COVID-19 pandemic as the rodents are one of the most widespread groups of mammals and live with human populations quite closely. It remains to be clarified if the extension of host range and immune evasion is co-incidence or co-evolution. Moreover, as the consequence of mutation of RBD is so intensive, the monitoring of mutations in RBD is important not only for predicting possible immune evasion and infectivity enhancement but also predicting novel host range for possible bi-directional anthroponosis/zoonosis, which could lead to the modification of prevention policy to block the possible new cross-species transmission route.

Our study also indicates that the common standard laboratory mice could serve as the models for SARS-CoV-2 infection and pathogenesis. In contrast to the mouse-adapted viral strains which have acquired some mutations not found in human epidemic strains and may not be able to infect, replicate and induce pathogenesis in humans as epidemic strains do, SARS-CoV-2 (B.1.351) is one of the major variant epidemics in many countries. Alternatively, many transgenic hACE2 mice, which can be infected by natural viral strains, have been specifically generated utilizing various genetic modifications. The human *ACE2* gene is introduced into the mouse genome under the control of a tissue-specific promoter,^17,21^ mouse *ACE2* promoter, or universal promoter.^20,22^ Instead of permanent genetic modification, some groups also develop hACE2-transduced mouse models, which are generated by intratracheally transducing mice with Ad5 or AAV encoding for hACE2.^18,19,23^ However, hACE2 in these mice is ectopically expressed. We cannot rule out the possibility that ectopic expression of hACE2 may change the tissue tropism of SARS-CoV-2 and the route for innate and acquired immune responses, which cannot reflect COVID-19 pathogenesis. Our models reported here are superior to the above models because the genetic modifications are not necessary for viruses or mice. The B.1.351-infected BALB/c and C57BL/6 mice showed similar symptoms as severe COVID-19 patients. B.1.351 infection in mice resulted in efficient virus replication with high titers in lungs and tracheas, severe pathological lung lesions, and inflammatory responses with elevated IL-6 and IL-10 expression. We also found low levels of virus in several other tissues, including the heart, liver, intestine, and lymphoid tissues. However, we did not detect viral RNA copies and pathological changes in the brains of B.1.351-infected young mice. Viral RNAs in brain tissue were only found in a few cases of deceased COVID-19 patients (most of which were the elderly with ages ranging from 51 to 94 years) and SARS-CoV-2-infected hACE2 transgenic mice.^21,56^ SARS-CoV-2 might infiltrate the brains of immunocompromised individuals or lethal-infected mice. Nevertheless, we cannot rule out the possibility that different SARS-CoV-2 variants might contribute to divergent viral tropisms. Collectively, B.1.351 variant utilizes mACE2 as the receptor to infect mice, and wild-type laboratory mice are inexpensive and easily genetically manipulated, which not only provides inexpensive and accessible biological materials but also provides simple mouse models to mimic SARS-CoV-2 infection and replicate symptoms of COVID-19.

## Materials and methods

### Plasmid construction

Different Spike protein-expressing plasmids, which were derived from eight SARS-CoV-2 lineages, were constructed to package pseudotyped SARS-CoV-2 S/HIV-1 viruses. The gene encoding the Spike protein of the original D614 virus (Wuhan-Hu-1, GISAID: EPI_ISL_402125) was codon-optimized, followed by cloning into pcDNA3.1-MCS-IRES-eGFP-WPRE vector. The gene encoding the Spike protein of the G614 virus (SYSU-IHV, EPI_ISL_444969) was generated by introducing the D614G mutation into the Spike protein of the D614 virus utilizing site-directed mutagenesis. Genes encoding Spike proteins of B.1.1.7 (GISAID: EPI_ISL_581117), B.1.351 (EPI_ISL_678597), P.1 (EPI_ISL_792683), B.1.429 (EPI_ISL_1675148), B.1.525 (EPI_ISL_1093465), and B.1.617.1 (EPI_ISL_1372093) were constructed by multiple rounds of overlapping polymerase chain reaction (PCR) to introduce specific mutation combinations. The Spike protein of B.1.1.7 harbored 9 mutations, which included ΔH69/V70, ΔY144, N501Y, A570D, D614G, P681H, T716I, S982A, and D1118H. The Spike protein of B.1.351 harbored 9 mutations, including L18F, D80A, D215G, R246I, K417N, E484K, N501Y, D614G, and A701V. The Spike protein of P.1 harbored 7 mutations: K417T, E484K, N501Y, D614G, H655Y, T1027I, and V1176F. The Spike protein of B.1.429 contained 4 mutations: S13I, W152C, L452R, and D614G. The Spike protein of B.1.525 contained 5 mutations: Q52R, E484K, D614G, Q677H, and F888L. The Spike protein of B.1.617.1 contained 8 mutations: G142D, E154K, L452R, E484Q, D614G, P681R, Q1071H, and H1101D. The Spike protein of B.1.617.2 contained 9 mutations: T19R, G142D, E156G, Δ157/158, L452R, T478K, D614G, P681R, and D950N. The Spike protein of C.37 contained 9 mutations: G75V, T76I, R246N, S247I, ΔD253, L452Q, F490S, D614G, and T859N. The sequences of all the Spike protein-expressing plasmids were verified by Sanger sequencing.

### Pseudotyped virus infection assay

Pseudotyped SARS-CoV-2 S/HIV-1 viruses were packaged in HEK293T cells by co-transfecting with a lentiviral construct pHIV-Luciferase (Addgene plasmid # 21375), a packaging construct psPAX2 (Addgene plasmid # 12260), and a plasmid expressing Spike protein of various variants, which included D614 virus, G614 virus, B.1.1.7, B.1.351, P.1, B.1.429, B.1.525, B.1.617.1, B.1.617.2, and C.37. The luciferase gene that was integrated into the pHIV-Luciferase construct can be expressed upon pseudotyped virus infection. The culture medium was exchanged with fresh Dulbecco’s modified Eagle’s medium (DMEM) supplemented with 10% fetal bovine serum and 1% penicillin–streptomycin 6 h post transfection. The supernatant that contained pseudotyped viruses was collected 48 h post transfection and filtered through 0.45 μm filters. The amount of pseudotyped viruses was determined by quantitative reverse transcription PCR (RT-qPCR) against the long-term repeat.^57^ Briefly, qPCR was performed with SYBR Premix ExTaq II Kit (Takara) by following the manufacturer’s instructions. The amounts of HIV-1 RNAs were determined by real-time qRT-PCR with the primer pair F (5’-CTGGCTAACTAGGGAACCCACTGCT-3’) and R (5’-GCTTCAGCAAGCCGAGTCCTGCGTC-3’). An in vitro-synthesized HIV-1 RNA, after quantification, was used as the external control for measuring the virion-associated viral RNA. HEK293T-hACE2 cells and HEK293T-mACE2 cells were generated as we described previously.^58^ Briefly, the endogenous hACE2 in HEK293T cells was knocked out with CRISPR-CAS9 technique. hACE2- and mACE2-expressing plasmids were transfected into hACE2 knock-out HEK293T cells to generate HEK293T-hACE2 and HEK293T-mACE2 cells, respectively. Both western blot and flow cytometry with antibodies against hACE2 and mACE2 were conducted to ensure the expression and quantitation of both proteins. To quantify infectivity of different pseudotyped viruses, various spike protein-packaged pseudotyped viruses were incubated with HEK293T-hACE2 and HEK293T-mACE2 cells that have knocked out endogenous hACE2. Cells were washed with phosphate-buffered saline (PBS) and lysed with passive lysis buffer 48 h post infection. The amounts of luciferase within different lysates were measured and represented as relative luminescence units utilizing luminometer (Promega). The normalized infectivity was calculated by normalizing relative luminescence units to viral titers.

### Surface plasmon resonance

The binding affinities of different RBD or S1 mutants to hACE2 and mACE2 were measured by SPR with a BIAcore^TM^ T100 instrument (GE Healthcare). Two Biacore CM5 sensor chips and an amine coupling kit were purchased from GE Healthcare. The suitable pH value of 4.5 for hACE2 and mACE2 immobilization was determined. The CM5 sensor chips were activated and then injected with hACE2 and mACE2 (2 μg/ml each, in 10 mM acetate buffer, pH 4.5) for 7 min. The residual activated groups on the surfaces were blocked with an injection of ethanolamine HCl (1 M) for 7 min. RBD or S1 mutants derived from D614 virus, G614 virus, B.1.1.7, B.1.351, P.1, and B.1.617.1 were diluted into different concentrations and then injected (30 μl/min). hACE2- and mACE2-bound proteins were monitored for about 120 s for each RBD or S1 mutant. The dissociation time was 200 s with running buffer per cycle. The association rate (“on rate,” *K*_a_) and dissociation rate (“off rate,” *K*_d_) were measured, followed by the calculating of the equilibrium dissociation constant (“binding constant,” *K*_D_).

### Animal models

Transgenic hACE2 mice (C57BL/6) were purchased from GemPharmatech Co., Ltd (Cat No.: T037657). These C57BL/6 background mice were generated via the CRISPR-CAS9 technique. Specific pathogen-free (SPF) 8-week old BALB/c mice and C57BL/6 mice were purchased from Guangdong Medical Laboratory Animal Center. All mice were housed in SPF facilities at Laboratory Animal Center of Sun Yat-sen University. Animal experiments were carried out in strict compliance with the guidelines and regulations of the Laboratory Monitoring Committee of Guangdong Province of China, and were approved by Ethics Committee of Zhongshan School of Medicine (ZSSOM) of Sun Yat-sen University on Laboratory Animal Care (Assurance Number: SYSU-IACUC-2021-B0020).

### Authentic SARS-CoV-2 infection

The SARS-CoV-2 (B.1.351) strain, named as 19nCoV-CDC-Tan-GDPCC, was isolated from a South African traveler and amplified twice with Vero E6 cells to propagate SARS-CoV-2 by the Guangdong Center for Disease Control in January 2021. After we obtained the virus tock, we amplified this strain again with Vero E6 cells. Therefore, the isolated 19nCoV-CDC-Tan-GDPCC virus was only passaged three times with Vero E6 cells. The sequences of both virus stock (second generation) and amplified virus (third generation) were confirmed by us. B.1.351 virus stocks were obtained from the supernatant of infected Vero E6 cells after incubation for 48 h. The viral titers were determined by a focus reduction neutralizing test targeting nucleocapsid (N) protein as we described previously.^28^ Eight-week-old SPF, transgenic hACE2 mice (C57BL/6 background), BALB/c mice, and C57BL/6 mice were intranasally challenged with different amounts (5 × 10^5^ FFU, 1 × 10^4^ FFU, 1 × 10^5^ FFU, or 1 × 10^6^ FFU) of B.1.351 virus (GDPCC-nCOV-84) after anesthetizing with isoflurane in BSL-3 facility. Authentic SARS-CoV-2 challenge studies were approved by the Ethics Committee of ZSSOM of Sun Yat-sen University on Laboratory Animal Care (Assurance Number: SYSU-IACUC-2021-B0020). Each group at each time point was assigned with four mice. Four C57BL/6 mice that were unchallenged were assigned to the mock group. Littermates of the same sex were randomly assigned to the uninfected and infected groups. Sera, lungs and other major organs including heart, liver, spleen, kidney, intestine, lymph node, and trachea were collected on Days 0, 1, 2, 3, 5, and 8 post infection. One part of these tissues proceeded to viral RNA extraction, one part was fixed with 4% paraformaldehyde buffer, and the others proceeded to cytokine and chemokine detection.

### Quantitative reverse transcription PCR

Different tissues of each mouse, including lung, heart, liver, spleen, kidney, intestine, lymph node, and trachea were collected and homogenized with gentleMACS M tubes (Miltenyi Biotec, 130-093-236) in a gentleMACS dissociator (Miltenyi Biotec, 130-093-235). Total RNAs of homogenized tissues were extracted with RNeasy Mini Kit (QIAGEN, 74104) according to the manufacturer’s instruction, followed by the qRT-PCR to determine the viral RNA copies of different tissues utilizing one-step SARS-CoV-2 RNA detection kit (PCR-Fluorescence Probing) (Da An Gene Co., DA0931). The SARS-CoV-2 *nucleocapsid (N)* gene was cloned into a pcDNA3.1 expression plasmid and in vitro transcribed to obtain RNAs for generating a standard curve. Indicated copies of *N* gene standards were 10-fold serially diluted and proceeded to RT-qPCR utilizing the same one-step SARS-CoV-2 RNA detection kit to obtain a standard curve. Reactions were carried out on a QuantStudio 7 Flex System (Applied Biosystems) according to the manufacturer’s instruction under the following reaction conditions: 50 °C for 15 min, 95 °C for 15 min, and 45 cycles of 94 °C for 15 s and 55 °C for 45 s. The viral RNA copies of each tissue were calculated into copies per ml and presented as log10 scale. The *N*-specific primers and probes were: N-F (5’-CAGTAGGGGAACTTCTCCTGCT-3’), N-R (5’-CTTTGCTGCTGCTTGACAGA-3’) and N-P (5’-FAM-CTGGCAATGGCGGTGATGCTGC-BHQ1-3’). During each RT-qPCR experiment, both a positive control and negative control of simulated RNA virus particles were included to monitor the entire experimental process and ensure the reliability of the test results.

### Histopathology and IHC

SARS-CoV-2 B.1.351-challenged mice, as well as uninfected mice, were euthanized in BSL-3 facility. Major tissues including lung, heart, liver, spleen, kidney, intestine, lymph node, and trachea were collected and fixed in 4% paraformaldehyde buffer for 48 h, followed by embedding with paraffin. All the samples proceeded to histopathology and IHC analysis (Nanjing FreeThinking Biotechnology Co., Ltd). Transverse sections were performed for the intestine. Longitudinal sections were performed for all the other tissues. All the sections were stained with HE. For IHC, lung sections of each mouse were deparaffinized and rehydrated with xylene and gradient alcohol. The antigen was microwave-retrieved by citric acid buffer (pH 6.0) and then quenched for endogenous peroxidases with 3% H_2_O_2_ for 10 min. Bovine serum albumin (BSA) was used to block non‑specific binding sites at room temperature for 30 min. The sections were incubated with rabbit anti-SARS-CoV-2 Nucleoprotein (N) at 1:5000 dilution for 24 h at 4 °C. Subsequently, the sections were incubated with goat anti-rabbit IgG secondary antibody (horseradish peroxidase (HRP) conjugated) for 2 h at room temperature and stained by 3,3’‑diaminobenzidine. Finally, the sections were dyed with hematoxylin, dehydrated with graduated concentrations of ethanol, cleared with xylene, and covered with neutral balsam for microscopic examination. Images were captured with HS6 (Sunny Optical Technology Co., Ltd) microscope.

### Cytokine and chemokine analysis

Total RNAs of the homogenized lungs were extracted with the RNeasy Mini Kit (QIAGEN, 74104), followed by reverse transcription with the PrimeScript RT reagent Kit (Takara). Reverse-transcribed cDNAs were proceeded to quantify the expression of target genes with SYBR Ex-taq premix (Takara) in a CFX96 Real-time PCR Detection System (Bio-Rad). Mouse *GAPDH* mRNA was measured as the internal control. Target cytokine genes included *Il1b*, *Il5*, *Il6*, *Il9*, *Il10*, *Il12a/Il12p70*, *Il17a*, *Ifng*, *Ccl4*, *Ccl5*, *Tnfa*, *Gcsf*, *Cxcl1*, *Gmcsf*, *Il1a* and *Il3* (Supplementary Table 1). The qPCR results were normalized to mouse *GAPDH*. The relative expression of each gene was calculated as 2^−ΔΔCt^ method. At least three independent experiments were analyzed. The primers used for RT-qPCR are listed in Supplementary Table 1. Cytokine proteins in sera were also quantified by a specific gene enzyme-linked immunosorbent assay kit (Beijing 4A Biotech Co., Ltd). These cytokine proteins included IL-6, IL-10, IL-1α, IL-1β, and CCL5.

### Serum IgG and IgM detection

Full-length His-tagged SARS-CoV-2 S protein (Sino Biological Inc., 40589-V08B1) (5 μg/ml) was coated on a Costar Stripwell^TM^ Microplate at 4 °C overnight (50 μl/well). The plates were blocked with 5% non-fat milk in PBS for 2 h at 37 °C. Subsequently, the plates were washed three times with PBS containing 0.1% v/v Tween-20 (PBS-T), followed by incubation with 10-fold serially diluted serum from mice in PBS-T for 1 h at 37 °C. After washing three times with PBS-T, A 1:10,000 dilution of HRP-conjugated goat anti-mouse IgG antibody or HRP-conjugated goat anti-mouse IgM antibody was incubated in each well for 1 h at 37 °C. The plates were then washed four times with PBS-T, followed by adding 100 μl of tetramethylbenzidine substrate (Invitrogen) at room temperature in the dark. After 10 min of incubation, the reaction was stopped with 100 μl 2 M H_2_SO_4_ solution. The absorbance of each well was measured at 450 nm.

### Plaque forming assay

The method was consistent as described previously.^28^ Briefly, the Vero E6 cells were seeded in 96-well plates at a density of 2 × 10^4^ cells per well and incubated the plates until cells reached 90–100% confluency. The tissue sample of BALB/c mice, hACE2 mice, and C57 mice was first ground in PBS buffer and then centrifuged, and the supernatant of 100μl was added to the Vero E6 cell culture for 1 h incubation at 37 °C. Then cell culture medium was removed from the 96-well plate; the DMEM containing 1.6% CMC was added to each well incubated for 24 h after the supernatant was removed. On the next day, the supernatant and cells were fixed with 200 μl of 4% paraformaldehyde in each well. After further incubation at 4 °C for 12 h, the plates were washed with 200 μl PBS each well for 3 times. And then, 100 μl PBS containing 0.2% Triton X-100 and 1% BSA was added to each well. After reaction for 30 min at room temperature, each well was washed with 200 μl PBS for 3 times and incubated 50 μl of the diluted primary antibody (Anti-SARS-N; 40143-T62-100), which was diluted to 1:1000 with PBS solution containing 1% BSA at 37 °C for 1 h. After primary antibody incubation, the cell within each well was washed 3 times with 200 μl PBS/T (0.1% Tween). The secondary antibody (goat anti-rabbit IgG HRP; SSA004-1) was diluted to 1:2000 with PBS solution containing 1% BSA. In all, 50 μl of the diluted secondary antibody was added to each well and incubated at 37 °C for 1 h, followed by washing with PBS/T three times. Fifty microliters of TrueBlue (KPL) were added to each well and set for 5 min shaking at room temperature. The plates were washed with ddH_2_O, and the liquid was eradicated, followed by spot counting using the ImmunoSpot Microanalyzer.

### Statistical analysis

All the measurements have been performed at least three times by at least two laboratory technicians. Detailed statistical information for specific experiments, including statistical tests, number of samples, mean values, standard errors of the mean (SEM), and *p* values derived from the indicated test, had been annotated in the main text and figure legends and shown in the figures. Statistical analysis was conducted with Graphpad Prism 8.0 or Microsoft Excel. Triplicate, quadruplicate, sextuplicate, and other replicate data are presented as mean ± SEM. A value of *p* ≥ 0.05 was considered to be not statistically significant and represented as “ns”. A value of *p* < 0.05 was considered to be statistically significant and represented as an asterisk (*). Value of *p* < 0.01 was considered to be more statistically significant and represented as double asterisks (**). Value of *p* < 0.001 was considered to be the most statistically significant and represented as triple asterisks (***). Value of *p* < 0.0001 was considered to be extremely statistically significant and represented as quadruple asterisks (****). For comparing mean differences between groups that were split on one independent variable, one-way analysis of variance (ANOVA) with Tukey’s multiple comparison test was used. For comparing mean differences between groups that were split into two independent variables, two-way ANOVA with Tukey’s multiple comparisons test or Dunnett’s multiple comparisons test was used. For comparing four groups of survival data, Logrank test was used.

## Supplementary information

Supplementary figure 1-6

## Data Availability

All supporting data have been included in the manuscript and the Supplementary Files. Further information and requests for resources and reagents should be directed to and will be fulfilled by the corresponding authors, X.M. (maxc6@mail.sysu.edu.cn) and H.Z. (zhangh92@mail.sysu.edu.cn).

## References

[1] Zhu N (2020). A novel coronavirus from patients with pneumonia in China, 2019. N. Engl. J. Med..

[2] Meng B (2021). Recurrent emergence of SARS-CoV-2 spike deletion H69/V70 and its role in the Alpha variant B.1.1.7. Cell Rep..

[3] Tegally H (2021). Detection of a SARS-CoV-2 variant of concern in South Africa. Nature.

[4] Candido DS (2020). Evolution and epidemic spread of SARS-CoV-2 in Brazil. Science.

[5] Ozer, E. A. et al. High prevalence of SARS-CoV-2 B.1.1.7 (UK variant) and the novel B.1.525 lineage in Oyo State, Nigeria. Preprint at *medRxiv*10.1101/2021.04.09.21255206 (2021).

[6] Zhou H (2021). B.1.526 SARS-CoV-2 variants identified in New York City are neutralized by vaccine-elicited and therapeutic monoclonal antibodies. mBio.

[7] Cherian S (2021). SARS-CoV-2 Spike Mutations, L452R, T478K, E484Q and P681R, in the Second Wave of COVID-19 in Maharashtra, India. Microorganisms.

[8] Kimura, I. et al. SARS-CoV-2 lambda variant exhibits higher infectivity and immune resistance. Preprint at *bioRxiv*10.1101/2021.07.28.454085 (2021).

[9] Muñoz-Fontela C (2020). Animal models for COVID-19. Nature.

[10] Mahdy, M. A. A., Younis, W. & Ewaida, Z. An overview of SARS-CoV-2 and animal infection. *Front. Vet. Sci*. **7**, 596391 (2020). 10.3389/fvets.2020.596391 PMC7759518 33363234

[11] Zhou P (2020). A pneumonia outbreak associated with a new coronavirus of probable bat origin. Nature.

[12] Sun S (2021). Characterization and structural basis of a lethal mouse-adapted SARS-CoV-2. Nat. Commun..

[13] Leist SR (2020). A mouse-adapted SARS-CoV-2 induces acute lung injury and mortality in standard laboratory mice. Cell.

[14] Dinnon KH (2020). A mouse-adapted model of SARS-CoV-2 to test COVID-19 countermeasures. Nature.

[15] Gu H (2020). Adaptation of SARS-CoV-2 in BALB/c mice for testing vaccine efficacy. Science.

[16] Rathnasinghe, R. et al. The N501Y mutation in SARS-CoV-2 spike leads to morbidity in obese and aged mice and is neutralized by convalescent and post-vaccination human sera. Preprint at *medRxiv*10.1101/2021.01.19.21249592 (2021).

[17] Winkler ES (2020). SARS-CoV-2 infection of human ACE2-transgenic mice causes severe lung inflammation and impaired function. Nat. Immunol..

[18] Israelow B (2020). Mouse model of SARS-CoV-2 reveals inflammatory role of type I interferon signaling. J. Exp. Med..

[19] Hassan AO (2020). A SARS-CoV-2 infection model in mice demonstrates protection by neutralizing antibodies. Cell.

[20] Bao L (2020). The pathogenicity of SARS-CoV-2 in hACE2 transgenic mice. Nature.

[21] Jiang R-D (2020). Pathogenesis of SARS-CoV-2 in transgenic mice expressing human angiotensin-converting enzyme 2. Cell.

[22] Sun S-H (2020). A mouse model of SARS-CoV-2 infection and pathogenesis. Cell Host Microbe.

[23] Sun J (2020). Generation of a broadly useful model for COVID-19 pathogenesis, vaccination, and treatment. Cell.

[24] Li Q (2021). SARS-CoV-2 501Y.V2 variants lack higher infectivity but do have immune escape. Cell.

[25] Hoffmann M (2020). SARS-CoV-2 cell entry depends on ACE2 and TMPRSS2 and is blocked by a clinically proven protease inhibitor. Cell.

[26] Hou YJ (2020). SARS-CoV-2 D614G variant exhibits efficient replication ex vivo and transmission in vivo. Science.

[27] Volz E (2021). Evaluating the effects of SARS-CoV-2 spike mutation D614G on transmissibility and pathogenicity. Cell.

[28] Ma X (2020). Nanoparticle vaccines based on the receptor binding domain (RBD) and heptad repeat (HR) of SARS-CoV-2 elicit robust protective immune responses. Immunity.

[29] Bai L (2021). Coinfection with influenza A virus enhances SARS-CoV-2 infectivity. Cell Res..

[30] Zhang S (2020). SARS-CoV-2 binds platelet ACE2 to enhance thrombosis in COVID-19. J. Hematol. Oncol..

[31] Schaefer I-M (2020). In situ detection of SARS-CoV-2 in lungs and airways of patients with COVID-19. Mod. Pathol..

[32] Wu Y (2020). Prolonged presence of SARS-CoV-2 viral RNA in faecal samples. Lancet Gastroenterol. Hepatol..

[33] Puelles VG (2020). Multiorgan and renal tropism of SARS-CoV-2. N. Engl. J. Med..

[34] Gavriatopoulou M (2020). Organ-specific manifestations of COVID-19 infection. Clin. Exp. Med..

[35] Buszko M (2021). Lessons learned: new insights on the role of cytokines in COVID-19. Nat. Immunol..

[36] Ragab D (2020). The COVID-19 cytokine storm; what we know so far. Front. Immunol..

[37] Tang Y (2020). Cytokine storm in COVID-19: the current evidence and treatment strategies. Front. Immunol..

[38] Del Valle DM (2020). An inflammatory cytokine signature predicts COVID-19 severity and survival. Nat. Med..

[39] Bao L (2020). Transmission of severe acute respiratory syndrome coronavirus 2 via close contact and respiratory droplets among human angiotensin-converting enzyme 2 mice. J. Infect. Dis..

[40] Damas J (2020). Broad host range of SARS-CoV-2 predicted by comparative and structural analysis of ACE2 in vertebrates. Proc. Natl Acad. Sci. USA.

[41] Liu Y (2021). Functional and genetic analysis of viral receptor ACE2 orthologs reveals a broad potential host range of SARS-CoV-2. Proc. Natl Acad. Sci. USA.

[42] Li R (2021). Differential efficiencies to neutralize the novel mutants B.1.1.7 and 501Y.V2 by collected sera from convalescent COVID-19 patients and RBD nanoparticle-vaccinated rhesus macaques. Cell. Mol. Immunol..

[43] Hoffmann M (2021). SARS-CoV-2 variants B.1.351 and P.1 escape from neutralizing antibodies. Cell.

[44] Wang P (2021). Antibody resistance of SARS-CoV-2 variants B.1.351 and B.1.1.7. Nature.

[45] Planas D (2021). Sensitivity of infectious SARS-CoV-2 B.1.1.7 and B.1.351 variants to neutralizing antibodies. Nat. Med..

[46] Zhou D (2021). Evidence of escape of SARS-CoV-2 variant B.1.351 from natural and vaccine-induced sera. Cell.

[47] Garcia-Beltran WF (2021). Multiple SARS-CoV-2 variants escape neutralization by vaccine-induced humoral immunity. Cell.

[48] Greaney AJ (2021). Comprehensive mapping of mutations in the SARS-CoV-2 receptor-binding domain that affect recognition by polyclonal human plasma antibodies. Cell Host Microbe.

[49] Rodrigues JPGLM (2020). Insights on cross-species transmission of SARS-CoV-2 from structural modeling. PLoS Comput. Biol..

[50] Liu K (2021). Cross-species recognition of SARS-CoV-2 to bat ACE2. Proc. Natl Acad. Sci. USA..

[51] Glatter KA, Finkelman P (2021). History of the plague: an ancient pandemic for the age of COVID-19. Am. J. Med..

[52] Griffin BD (2021). SARS-CoV-2 infection and transmission in the North American deer mouse. Nat. Commun..

[53] Fagre A (2021). SARS-CoV-2 infection, neuropathogenesis and transmission among deer mice: Implications for spillback to New World rodents. PLoS Pathog..

[54] Bosler EM (1984). Prevalence of the Lyme disease spirochete in populations of white-tailed deer and white-footed mice. Yale J. Biol. Med..

[55] Botten J (2003). Persistent Sin Nombre virus infection in the deer mouse (*Peromyscus maniculatus*) model: sites of replication and strand-specific expression. J. Virol..

[56] Matschke J (2020). Neuropathology of patients with COVID-19 in Germany: a post-mortem case series. Lancet Neurol..

[57] Zhang Y (2014). A novel HIV-1-encoded microRNA enhances its viral replication by targeting the TATA box region. Retrovirology.

[58] Zhang J (2021). The interferon-stimulated exosomal hACE2 potently inhibits SARS-CoV-2 replication through competitively blocking the virus entry. Sig. Transduct. Target. Ther..

