# Supplementary material for: Infection of wild-type mice by SARS-CoV-2 B.1.351 variant indicates a possible novel cross-species transmission route
Source: Signal Transduct Target Ther. 2021 Dec 14;6:420. doi: 10.1038/s41392-021-00848-1 (PMC8669038; doi:10.1038/s41392-021-00848-1)
Supplement: Supplementary file 1 — Supplementary figure 1-6 [file 41392_2021_848_MOESM1_ESM.pdf]

Supplementary Materials for

Infection of wildtype mice by SARS-CoV-2 B.1.351 variant indicates a possible novel cross-species transmission route

Ting Pan[#], Ran Chen[#], Xin He[#], Yaochang Yuan[#], Xiaohui Deng, Rong Li, Haiping Yan, Shumei Yan, Jun Liu, Yiwen Zhang, Xiantao Zhang, Fei Yu, Mo Zhou, Changwen Ke, Xiancai Ma* and Hui Zhang*

*Corresponding authors.

Xiancai Ma Tel: +86 185 8882 0419; E-mail: maxc6@mail.sysu.edu.cn

Hui Zhang Tel: +86 137 1063 5612; E-mail: zhangh92@mail.sysu.edu.cn

[#] These authors contributed equally to this work

This PDF file includes:

Supplementary Figures 1 to 6

Supplementary Fig. 1

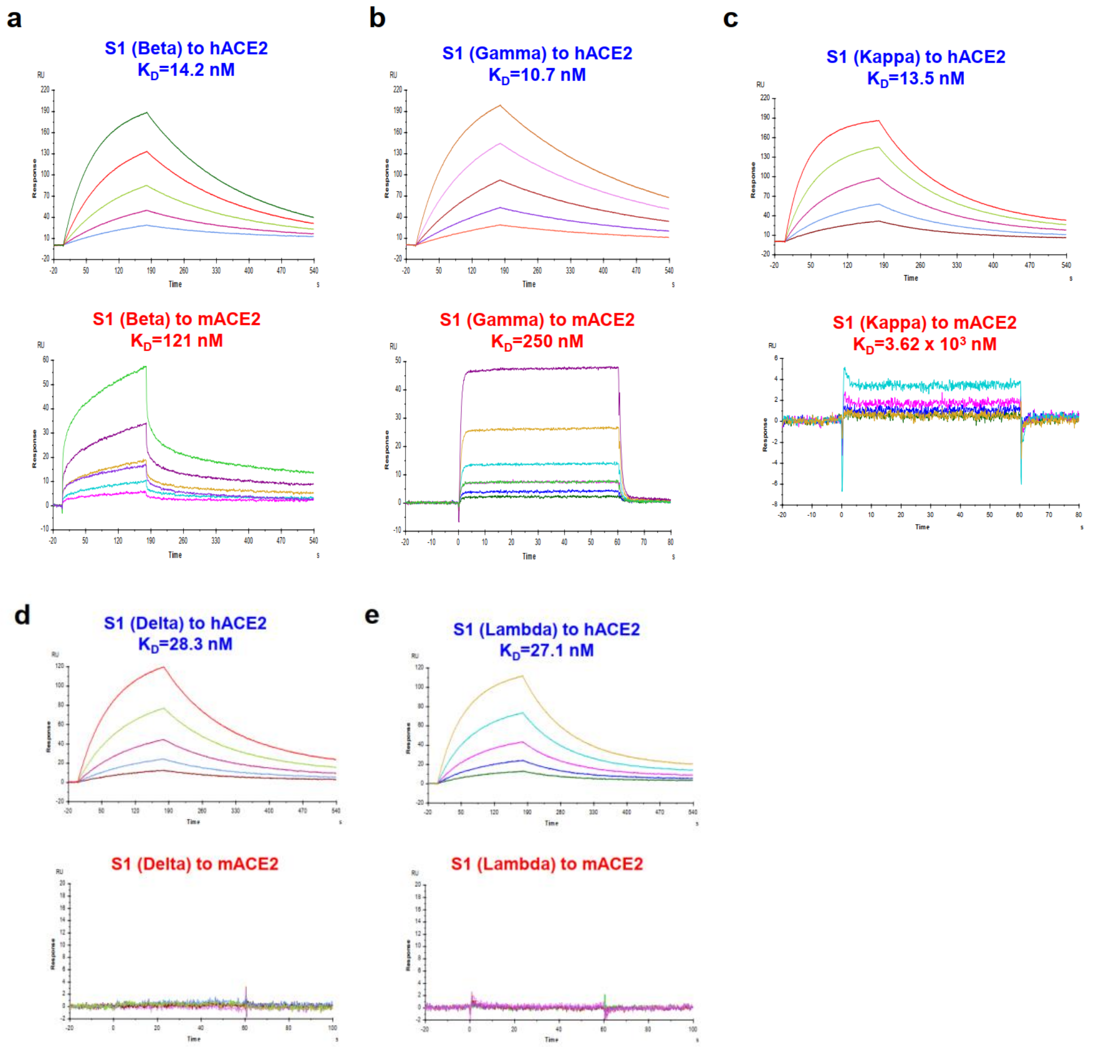

The binding affinities of S1 proteins to hACE2 and mACE2. The binding affinities of different S1 mutants to hACE2 and mACE2 were measured by surface plasmon resonance (SPR) assays with a Biacore^{TM} T100 instrument. The Ka, Kd and KD values were measured and calculated by the software BIAevaluation. The KD value shown was a mean of three independent experiments. These different S1 mutants included S1 (Beta) (**a**), S1 (Gamma) (**b**), S1 (Kappa) (**c**), S1 (Delta) (**d**), and S1 (Lambda) (**e**).

Supplementary Fig. 2

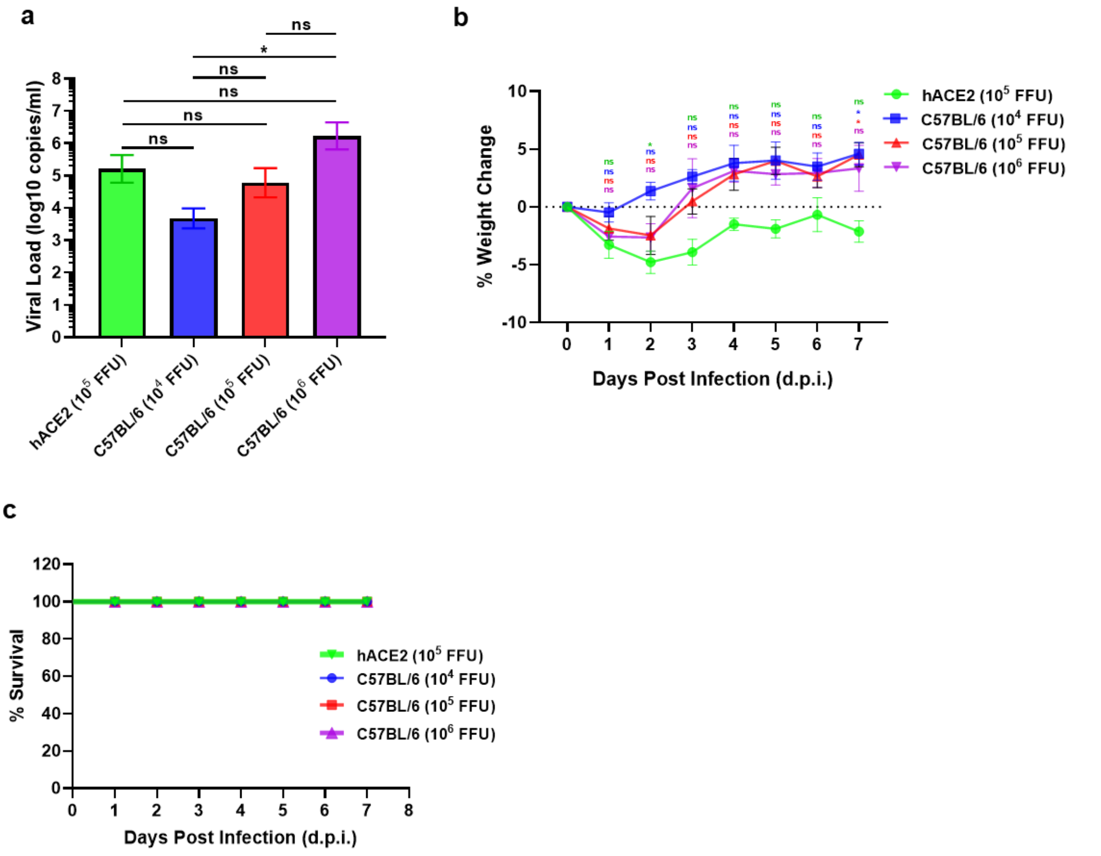

The different infective doses of B.1.351-infected mice. a, The hACE2 mice were intranasally challenged with 1×10^5 FFU of B.1.351 virus. C57BL/6 mice were challenged with 1×10^4 FFU, 1×10^5 FFU and 1×10^6 FFU of B.1.351 virus respectively. The viral RNA copies of lung and trachea samples of each mouse were quantitated on Day 7 and represented as log10 copies per ml (n=4). **b,** Weight changes of B.1.351-infected mice. The weight of each mouse in each time point was normalized to their initial weight (n=4). **c,** Survival data of B.1.351-infected mice, which was shown as Kaplan–Meier curve. Data represented as mean ± SEM in quadruplicate. Pvalues in (**a**) were calculated by one-way ANOVA with Tukey's multiple comparison test. P-values in (**b**) were calculated by two-way ANOVA with Dunnett's multiple comparisons test. Survival data in (**c**) was analyzed with Logrank test. ns = $p \geq 0.05$, *$p < 0.05$.

Supplementary Fig. 3

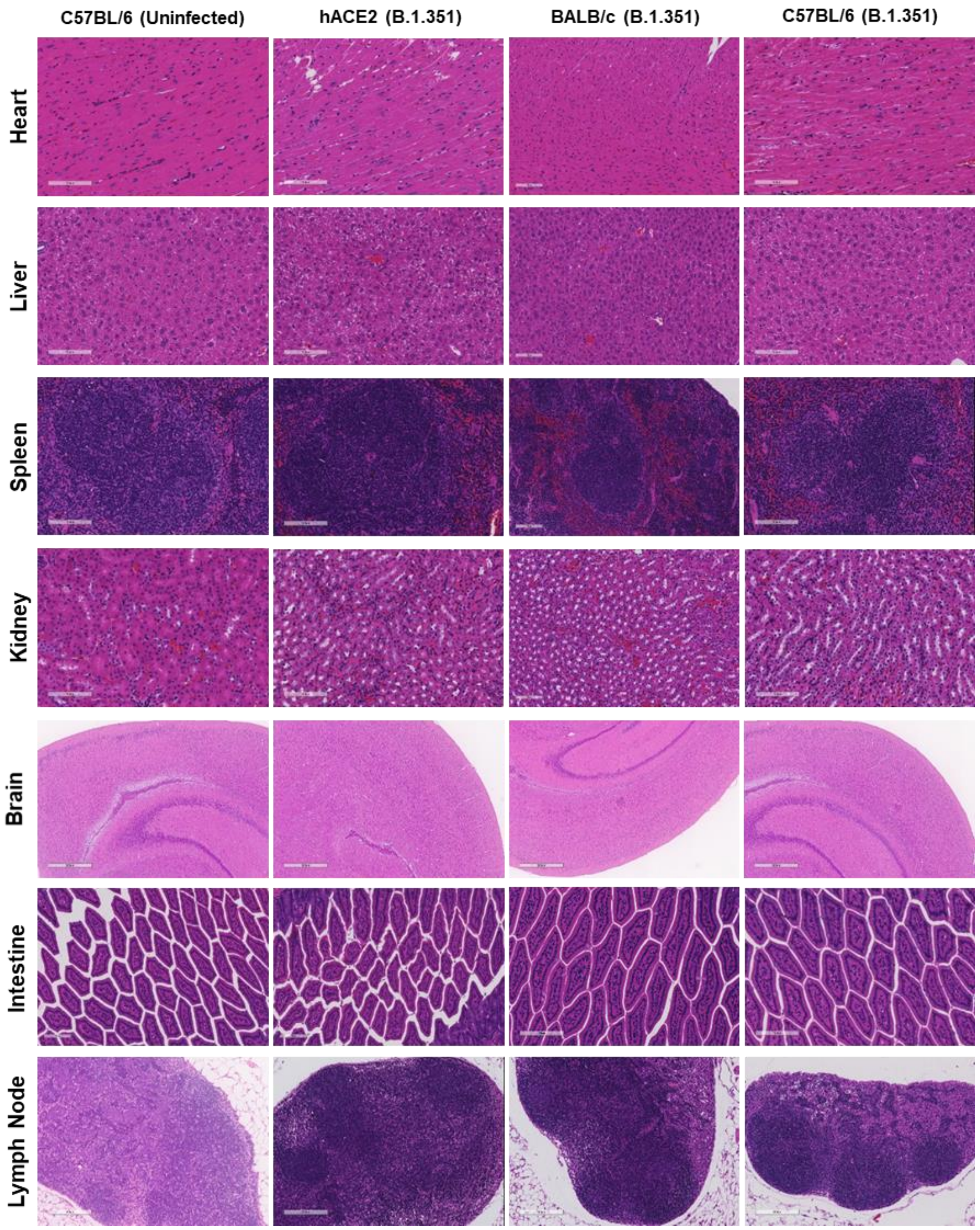

Histopathology analysis of B.1.351-infected mice. Hearts, livers, spleens, kidneys, brains, intestines and lymph nodes of each mice on Day 5 were fixed with 4% paraformaldehyde buffer and stained with HE. Scale bars for brains represented 500 μm. Scale bars for lymph nodes represented 200 μm. Scale bars for hearts, livers, spleens, kidneys and intestines represented 100 μm. Each picture was a representation from four mice.

Supplementary Fig. 4

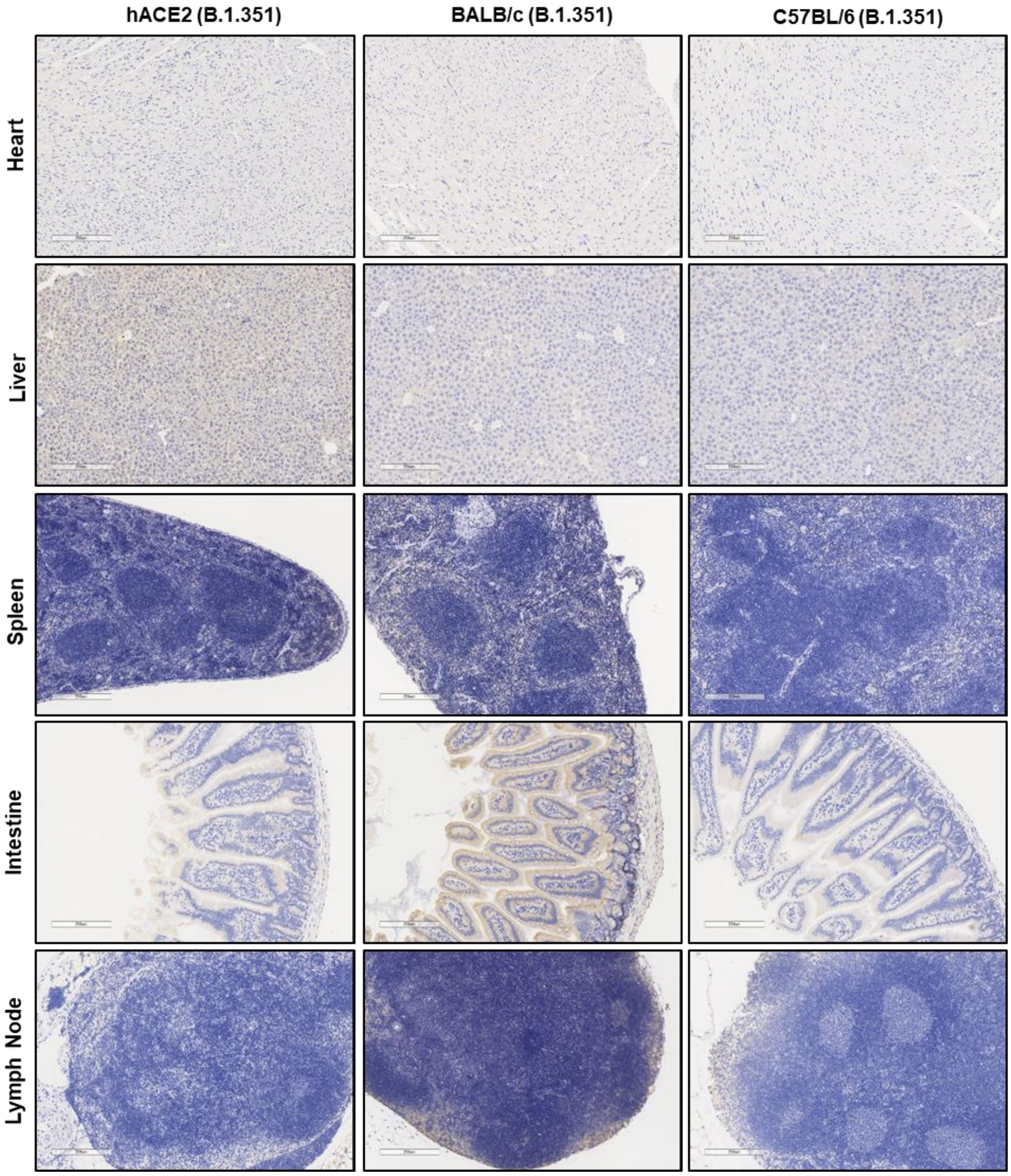

Immunohistochemistry analysis of B.1.351-infected mice. Hearts, livers, spleens, intestines and lymph nodes of B.1.351-infected mice on Day 2 were proceeded to IHC assays with antibodies against SARS-CoV-2 N proteins. Scale bars represented 100 μm. Each picture was a representation from four mice.

Supplementary Fig. 5

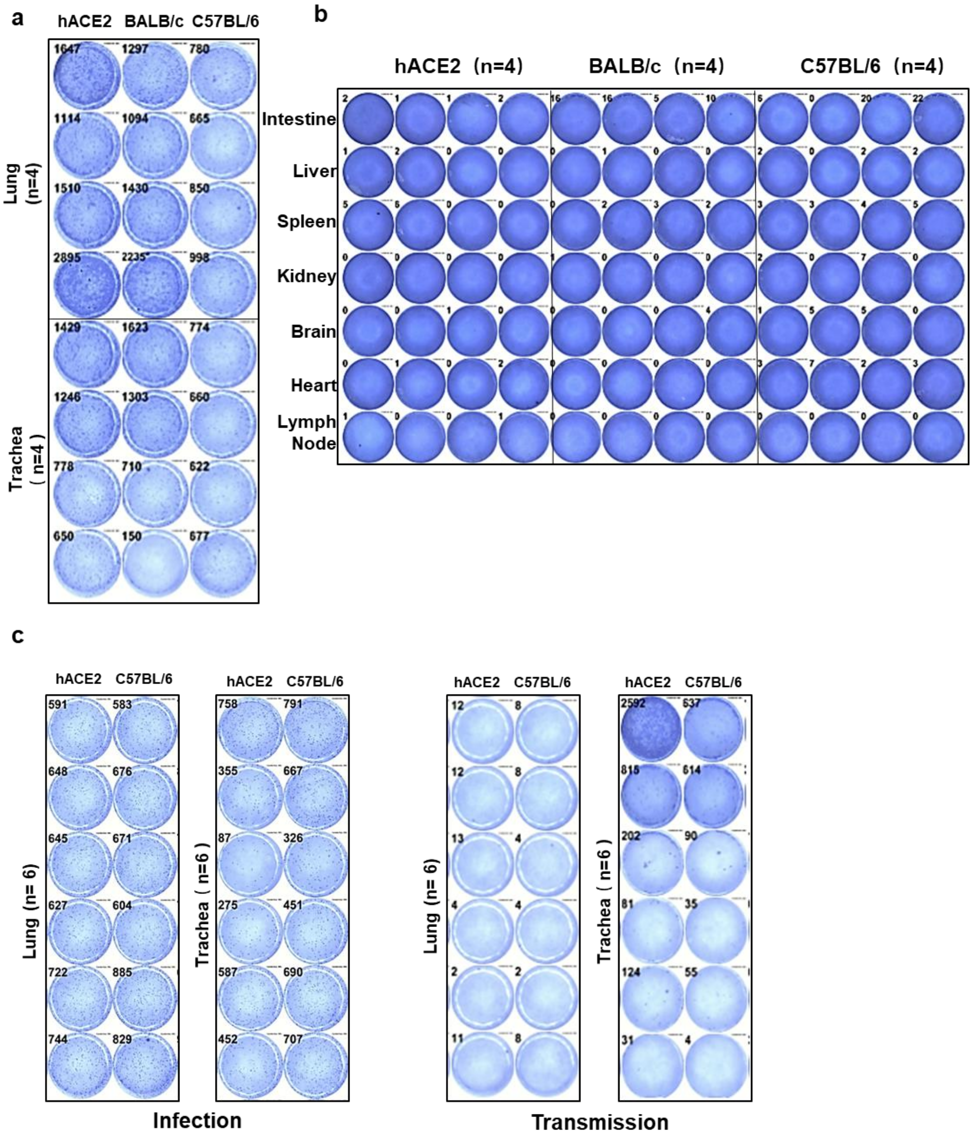

The plaque forming assay in tissue samples. a, The viral load of lung and trachea tissue samples of infected mice (from Fig.3) were determined by plaque forming assay with anti-SARS-CoV-2 N protein antibody staining. The representatives of spot wells within 1:100 dilution group were shown (n=4). **b,** The viral load of different tissue samples of infected mice (from Fig.3) were determined by plaque forming assay with anti-SARS-CoV-2 N protein antibody staining. The representatives of spot wells within 1:1 dilution group were shown (n=4). **c,** The viral load of lung and trachea tissue samples of infected mice (from Fig.5) were determined by plaque forming assay with anti-SARS-CoV-2 N protein antibody staining. The representatives of spot wells in infection group with 1:100 dilution group were shown and the representatives of spot wells in transmission group with 1:1 dilution group were shown.

Supplementary Fig. 6

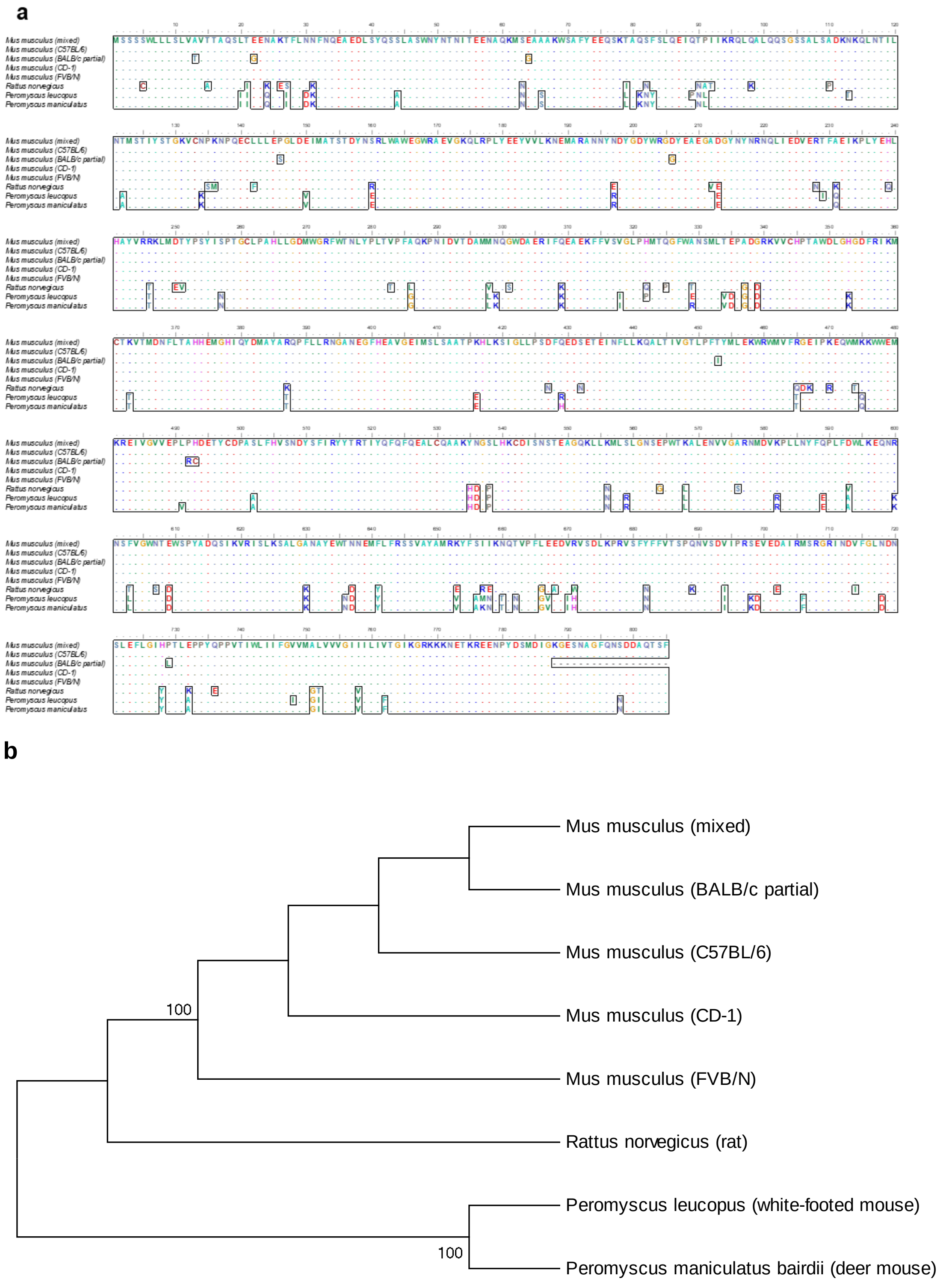

Homology analysis of ACE2 among selected rodents. a, Homology analysis of ACE2 among selected rodents which contained five *Mus musculus* strains (mixed strain, BALB/c (partial), C57BL/6, CD-1 and FVB/N), *Rattus norvegicus* (rat), *Peromyscus leucopus* (white-footed mouse) and *Peromyscus*

maniculatus bairdii (deer mouse). The alignments of sequences were built by using ClustalW method. All ambiguous positions were removed for each sequence pair. **b,** The evolutionary history was inferred using the Neighbor-Joining method based on the protein sequences of ACE2. The percentage of replicate trees in which the associated strains clustered together in the bootstrap test (1000 replicates) were shown next to the branches. The tree was drawn to scale, with branch lengths in the same units as those of the evolutionary distances used to infer the phylogenetic tree.
